# Research on calibrating rock mechanical parameters with a statistical method

**DOI:** 10.1371/journal.pone.0176215

**Published:** 2017-05-18

**Authors:** Zhen Liu, Ye Guo, Shuheng Du, Gengyu Wu, Mao Pan

**Affiliations:** 1 The Key Laboratory of Orogenic Belts and Crustal Evolution (MOE), School of Earth and Space Sciences, Peking University, Beijing, China; 2 Oil and Gas Institute, School of Earth and Space Sciences, Peking University, Beijing, China; 3 Petroleum Department, Cullen College of Engineering, University of Houston, Houston, Texas, United States of America; Beihang University, CHINA

## Abstract

Research on the modeling of rock mechanics parameters is of great significance to the exploration of oil and gas. The use of logging data with the Kriging interpolation to study rock mechanics parameters has been proven to be effective in reservoir prediction and other oilfield applications and can provide additional data. However, there will sometimes be a great deviation due to the limited samples and the strong heterogeneity of a layer. To solve this problem, a new approach was proposed to calibrate rock mechanical models through the statistical analysis of logging data. A module was developed to calibrate rock mechanics parameters automatically, which was then applied to the Wangyao area of the Ansai oilfield. This method significantly improved the accuracy of rock mechanics modeling.

## Introduction

In the exploration and development of lithological oil-gas reservoirs, rock mechanics parameters, such as Young’s modulus, Poisson’s ratio, the effective stress coefficient, and the angle of internal friction, are crucial to geo-stress simulation, wellbore stability analysis, sweet spot prediction, and simulation techniques. In recent years, as the use of unconventional resources have developed rapidly, high-accuracy rock mechanics modeling has drawn a great degree of attention and been studied by experts worldwide, and many research results have been put into field application.

Considering the unbiased optimal advantages, the Kriging method is one of the most widely used methods in rock mechanics modeling. However, due to some limitations of Kriging in practical applications, many other methods have been developed to improve the applicability of Kriging, such as simple Kriging, ordinary Kriging[1], co-Kriging[2], universal Kriging[3], and indicator Kriging[4]. In recent years, Roustant optimized the method to solve a covariance function by proposing a nonnegative solution of linear equations to eliminate part of the subjective impacts[5]. Erum[6] proves that Bayesian universal Kriging fits better than the universal Kriging in predicting sodium concentrations. Hu improved the results of Kriging, which can be impacted by scaling, with the Bayesian-based collocated co-Kriging method [7]. The above methods can improve the accuracy mathematically but fail to consider the spatial distributions of rock mechanics parameters and are unable to optimize the search radius and range. Therefore, these methods can cause a large error in the simulation results. To solve this problem, this study analyzed the behavior around the wellbore, constrained the Kriging results by setting up the law of geological parameters’ distribution characteristics, and improved the mechanics modeling of rock with a calibration method. The module was also developed to constrain the rock mechanics parameters. This proposed method was applied successfully to the Wangyao area of the Ansai oilfield, and the results showed that the accuracy of rock mechanics modeling improved significantly.

## Materials and methods

### Materials and characterization

The research reservoir is located in the mid-east region of the Ordos basin, the stratigraphic occurrence is gentle [8], the local structure is stable, and there are no major fault activities. The dipping magnitudes of the strata are approximately 0.5 degree; the average gradient is 8–10 m/km. Differential compaction effects form a low angle nose-like uplifted structure. The delta sand-mud interaction affected the accumulation of hydrocarbons in this area. The reservoir has low porosity and permeability but is highly fractured [9–11], which results in high heterogeneity and complexity in the distribution of rock mechanics properties [12]. Therefore, it is critical to create a high-resolution rock mechanics model based on the abundant well log data.

In this study, rock mechanics parameters at the wellbore were calculated using conventional logging data from more than 500 wells in the Wangyao area, together with some cross-dipole acoustic logging data (X-MAC). The file format of logging data is “.Las”; these data include the version information, using “~VERSION” as the identifier. Well information is separated by the "~WELL" logo. The curve information has a "~CURVE" flag; ASCII data are identified by "~A", which includes the actual data on logging.

### Structure of rock mechanics model

Traditional rock mechanics modeling fails to consider the whole spatial distribution trend of rock mechanics parameters by simply using the neighborhood properties and local locations. Therefore, this study restricted rock mechanics modeling by analyzing Young’s modulus and Poisson’s ratio at wellbores from the macroscopic view to achieve practical results.

**Fig 1:**
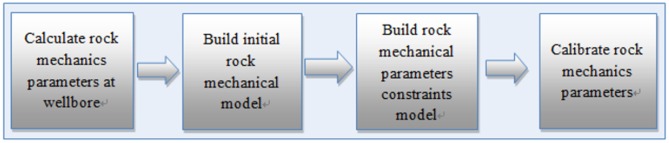
Rock mechanical modeling workflow.

This study consists of four parts (Fig 1). First, data were collected and rock mechanics parameters at wellbores were calculated, such as Young’s modulus and Poisson’s ratio, using correlation analysis and the regression approach to address cross-dipole acoustic logging and density logging. Then, the initial rock mechanics model was built using ordinary Kriging, including searching the neighborhood, solving the covariance function, and meshing. The original data for Kriging come from the rock mechanics parameters at wellbores. Statistical analyses of Young’s modulus and Poisson’s ratio were carried out. Then, a rock mechanics parameter restriction model was built to better calibrate the model. Finally, a module was developed with the programming language C++ to calibrate the Young’s modulus and Poisson’s ratio and improve the accuracy of the rock mechanical model.

### Calculation of rock mechanics parameters at the wellbore

The current methods to collect and calculate rock mechanics parameters are mature and mainly include the measurement method [13] and logging operation method [14]. During the exploration and development of oil fields, conventional logging plays an important role in extracting rock mechanics parameters due to its rich data, low cost, and higher accuracy than seismic data. It also contains important information, such as the reservoir structure, lithology, and physical properties.

In this study, rock mechanics parameters at the wellbore were calculated using X-MAC logging[15] together with density logging, and Young’s modulus and Poisson’s ratio were calculated with the following equations:

$$V=\frac{\Delta t_{s}^{2}-2\Delta t_{p}^{2}}{2\left(\Delta t_{s}^{2}-\Delta t_{p}^{2}\right)}$$ (1)

$$E=\frac{\rho }{\Delta t_{s}^{2}}\frac{3\Delta t_{s}^{2}-4\Delta t_{p}^{2}}{2\left(\Delta t_{s}^{2}-\Delta t_{p}^{2}\right)}$$ (2)

where V refers to Poisson’s ratio, dimensionless; E refers to Young’s modulus, MPa; ρ is the density, g/cm3; and Δt_s_ and Δt_p_ denote the shear wave slowness and compressional wave slowness, respectively.

**Fig 2:**
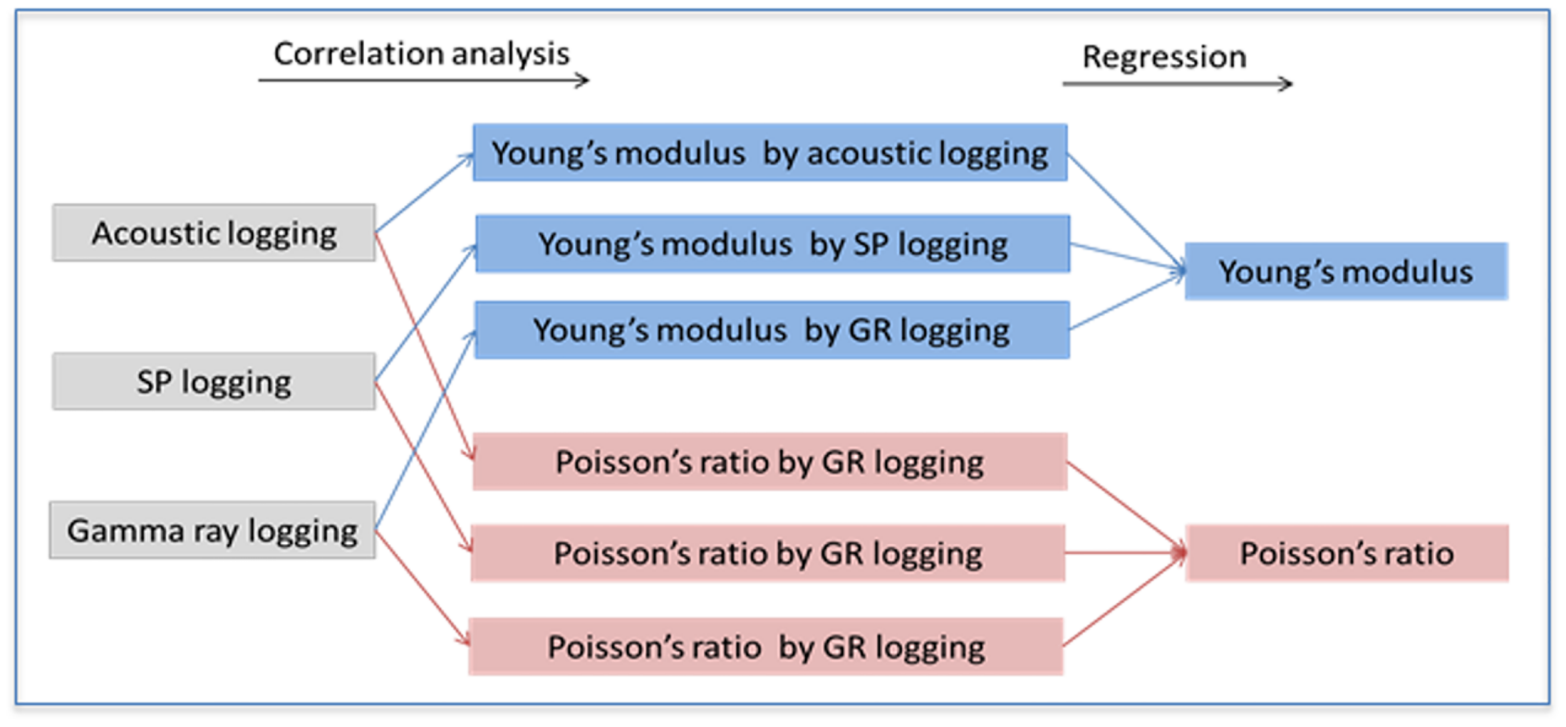
Rock mechanics parameters calculated by conventional logging data.

Using X-MAC logging data to calculate Young’s modulus and Poisson’s ratio brings feasibility to revising the final results, which were calculated with acoustic logging, gamma ray logging, and spontaneous logging, and eventually the Young’s modulus and Poisson’s ratio were established through a regression (Fig 2).

**Fig 3:**
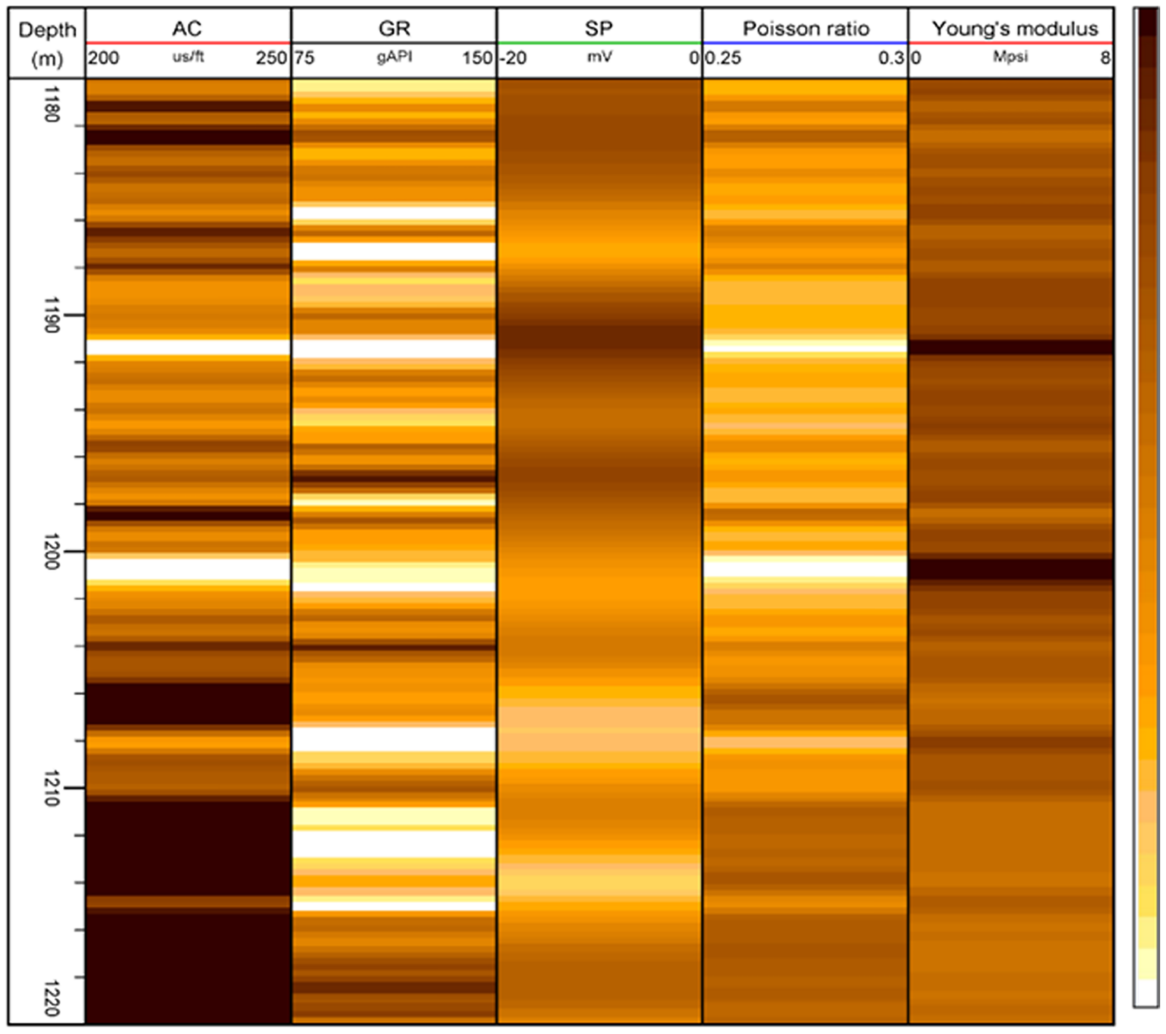
Correlation between conventional log curves and rock mechanics parameters in the Ansai oilfield.

According to the correlation analysis, there is a strong correlation between Young’s modulus/Poisson’s ratio and acoustic logging/gamma ray logging, which can bring good regression results. As shown in Fig 3, the value increases as the color scale darkens. Based on acoustic logging and gamma ray logging data, Young’s modulus and Poisson’s ratio were calculated with the following regression equations:

$$E=18.09731739-\left(519.51AC-1.157GR\right)/10^{3}$$ (3)

$$V=0.143566014+\left(536.869AC-9.18235GR\right)/10^{6}$$ (4)

where AC refers to the wave slowness, μs/m, and GR refers to the gamma ray data, API. The correlation coefficients from the above two equations are 0.996 and 0.998, respectively, which confirm the strong correlation between Young’s modulus/Poisson’s ratio and acoustic logging/gamma ray logging. After calculating the dynamic Young’s modulus and Poisson’s ratio of wells in the Wangyao area using the above equations, they were converted to static parameters using equations provided by Du [16]. Then, they can be used to change the stress field in a low-permeability reservoir and secondary exploitation.

### Constructing the initial rock mechanics model

Kriging interpolation is the core of geostatistics, and it is widely used in the field of geoscience. Kriging was first introduced by the South African mining engineer D.G. Krige in 1951 to resolve the problems of deposit reserve computation and error estimation [17]. Considerable research on Kriging has been published globally since then. The basic idea of Kriging is to predict the value by assigning weights to known sample points according to their spatial locations and correlations relative to the target point and calculating the moving weighted average of known values. The Kriging method takes advantage of property correlations and spatial locations between neighboring sample points, and it provides high interpolation estimation accuracy; therefore, this method has been used in the field of rock mechanics parameters modeling [18–21].

The theoretical model of the Kriging interpolation algorithm is expressed as follows:

$$G^{*}\left(x_{0}\right)=\sum_{k=1}^{n}\lambda_{i}\left(x_{k}\right)G\left(x_{k}\right)$$ (5)

where λ_i_(x_0_) are the weight coefficients, x is the location of the unknown point, x_k_ is the location of a known point, n is the number of measurement points that have a relationship with x, G*(x_0_) is the estimate of the unknown point, and G(x_k_) is the measurement value of the known point k. The equations for calculating weights λ_i_(x_0_) are as follows:

$$\{\begin{array}{c} \begin{array}{cc} \sum_{i=1}^{n}\gamma \left(x_{i}-x_{j}\right)\lambda_{i}+\mu =\gamma \left(x_{0}-x_{j}\right), & j=1,\ldots ,n \end{array}\\ \sum_{i=1}^{n}\lambda_{i}=1 \end{array}$$ (6)

where γ(x_i_−x_j_) is the variogram between known points i and j and μ is a Lagrange multiplier.

**Fig 4:**
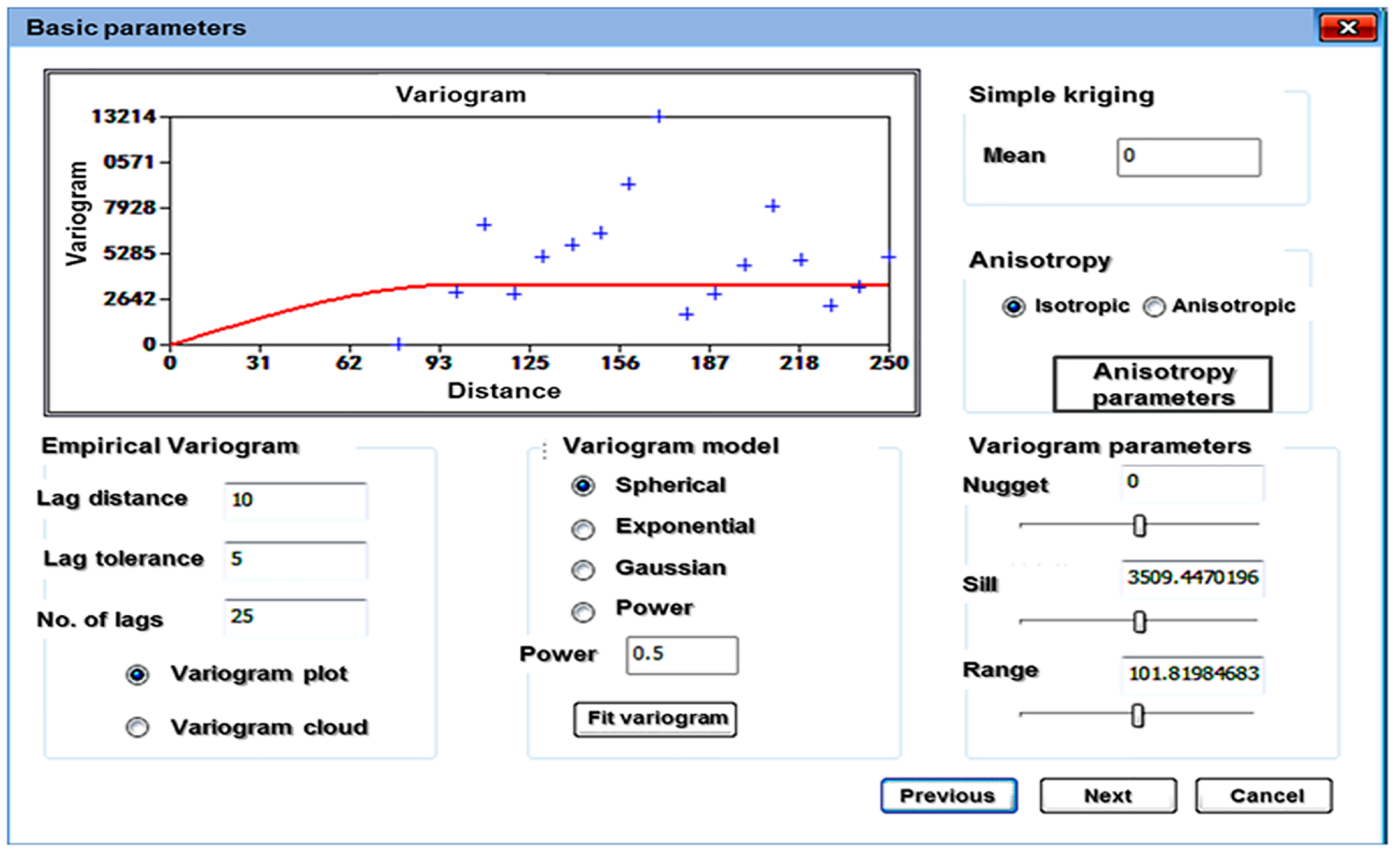
Fitting of variogram function.

**Fig 5:**
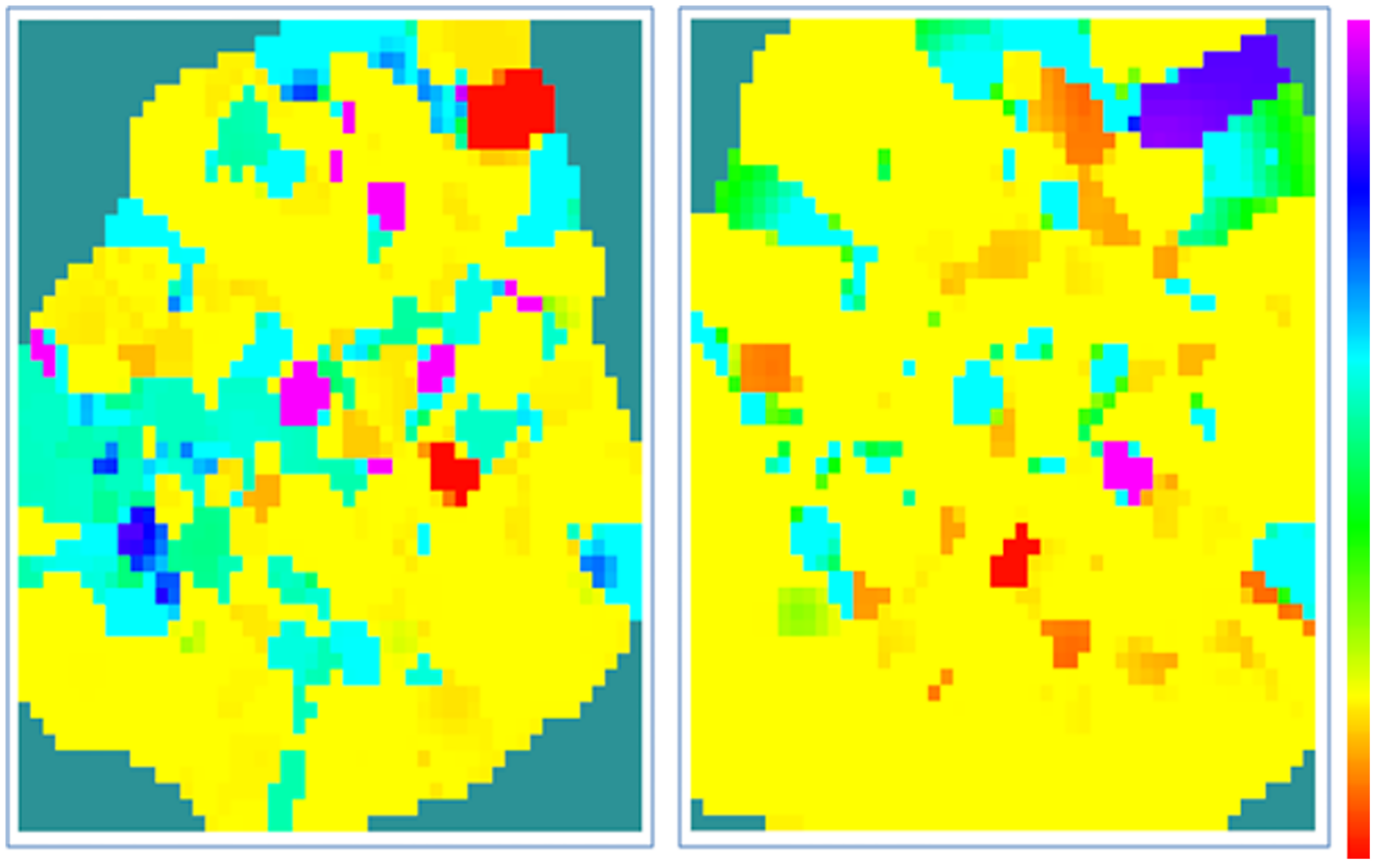
Distribution of rock mechanical parameters’. (A) Young’s modulus distribution. (B) Poisson’s Ratio distribution.

In this paper, the steps for using the Kriging method to construct rock mechanics parameters model were proposed and include inputting wellbore data, generating a 3D geo-model mesh, fitting the variogram model, searching the neighborhood data, and performing interpolation. The method for creating an initial rock mechanics model using Kriging is as follows:

1. Acquire the wellbore data. Wellbore rock mechanics parameters, including Young’s modulus and Poisson’s ratio, are calculated from conventional logging data and prepared in standard data formats for Kriging interpolation. In addition, due to the existence of abnormalities in log data, outliers will be processed or eliminated.
2. Construct the geological mesh. The stratigraphic model [22] is represented on a triangular surface mesh [23–28], but the Kriging interpolation of rock mechanics parameters is performed on volume mesh elements, so it is necessary to convert the triangular mesh geomodel into a volume mesh geomodel [29–32]. In this paper, the hexahedral element is selected as the basic unit, and each stratigraphic layer of the geomodel is constructed by mesh voxelization.
3. Fit the variogram model. Given the same source data and geomodel mesh, variograms influence the accuracy of Kriging interpolation. For layers with high heterogeneity, it is necessary to compute variograms in multiple directions to achieve higher interpolation accuracy. In this paper, the empirical variograms were computed with sample data. We select the spherical model as the variogram model to simplify the procedure and use least square regression to find the parameters in the variogram model. Fig 4 is a graphic interface of the variogram fitting function developed with the language C. Fig 4 shows that the spherical variogram is selected because it can be easily converted to a linear variogram to simplify calculation and because it is convenient for finding parameters such as nugget and sill [33–34]. The lag distance, lag tolerance and number of lags are required inputs for variogram fitting. Meanwhile, parameter isotropy or anisotropy is selected based on the actual scenario. The initial values of nugget, sill and range are calculated from the variogram fitting. These values are usually optimized according to the interpolation results. The Kriging calculation can be done after all required settings are provided.
4. Search the neighborhood. To calculate weights in Eq 6, it is necessary to search for grid cells in the neighborhood of the unknown point to obtain the Young’s modulus and Poisson’s ratio of the sample cell. However, when too many grid cells are found, the search efficiency is low; therefore, an algorithm was developed to find the closest grid cells to the point of interest to improve the modeling efficiency.
5. Interpolate using Kriging. After the weights λ_i_ are computed, Eq 5 is used to predict a weighted average value of unknown rock mechanics properties at a cell of interest, and then, an initial 3D rock mechanics model is constructed. As shown in Fig 5, where the left is 3D Young’s modulus distribution and the right is the Poisson’s ratio distribution. Low values are in purple, and high values are in red. It is certain that the rock mechanics properties are heterogeneous and vary significantly spatially.

### Building the rock mechanics parameter constraints model

There are more than 500 conventional wells in the whole Wangyao area. With this high well density, Young’s modulus and Poisson’s ratio at the wellbore can reflect the spatial distribution of rock mechanics properties, which provides a solid source to build a three-dimensional rock mechanical model. The Young’s modulus and Poisson’s ratio were analyzed mathematically and statistically, and they were used to calibrate the model. In this study, the statistical factors have been considered, including the maximum value, minimum value, average, variance, and probability distribution.

The variance, average and histogram of Young’s modulus and Poisson’s ratio at the wellbore and from the initial model were compared to better construct the theoretical model. Assume that the maximum, minimum, average, variance, frequency of the wellbore Poisson’s ratio and initial Poisson’s ratio are (*max*_*1*_, *min*_*1*_, *m*_*1*_, *σ*_*1*_, *t*_*1*_) and (*max*_*2*_, *min*_*2*_, *m*_*2*_, *σ*_*2*_, *t*_*2*_), respectively. The differences Δ*m* = *m*_1_ − *m*_2_ and Δ*σ* = *σ*_1_ − *σ*_2_ were calculated. Most importantly, the following linear approximation equation was utilized to optimize the distribution of initial rock mechanics parameters:

$$Pois_{1}\left(x\right)=k_{1}*\sqrt{\Delta \sigma }*Pois_{2}\left(x\right)+k_{2}*\Delta m$$ (7)

where Pois_1_(x) is the optimal Poisson’s ratio at location x; Pois_2_(x) is the initial Poisson’s ratio at location x; and k_1_ (k_1_>0) and k_2_ (k_2_>0) are coefficients.

## Results and discussion

### Development of calibration module

To calibrate the rock mechanics model efficiently, a module was developed to realize the automatic calculation of properties. This module consists of reading data from the model, defining the constraint model, calibrating the model and managing the data.

Due to the varying properties in a geological grid, such as lithological and geophysical properties, an efficient data reading module needs to be established that allows us to select different geophysical properties.

**Fig 6:**
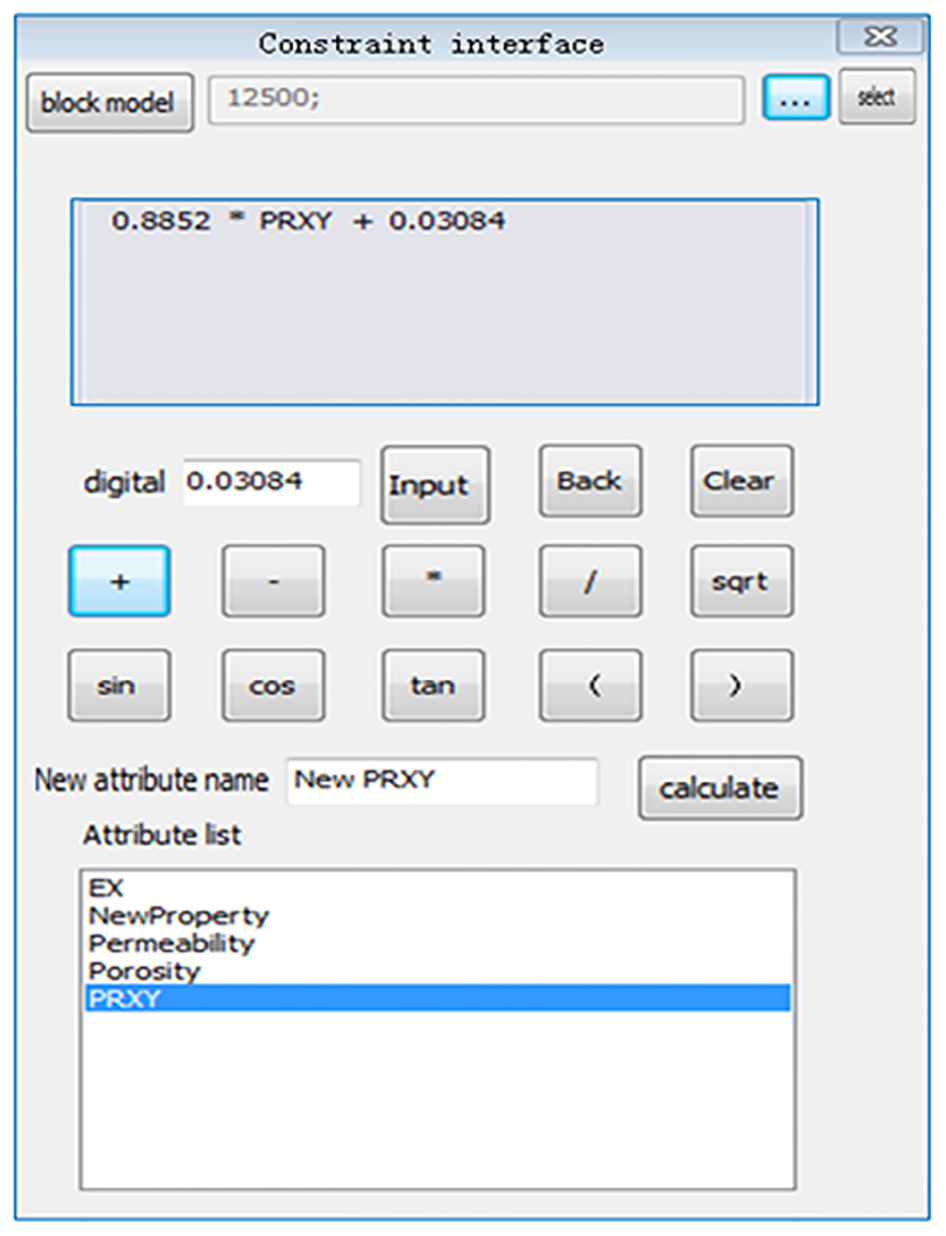
Interface of calibration model.

After loading the data from the last step, the calibration model was applied to modify all the properties. Different areas and different properties correspond to different calibration models. Therefore, the module was designed to customize the calibrated model so that users can input the coefficients of the model. This module also offers the feature of property extraction through the interface after calculation. Some simple calculations and value inputs are provided by the “property calculator” as shown in Fig 6, which can easily calibrate the model with a single property or multiple properties.

**Fig 7:**
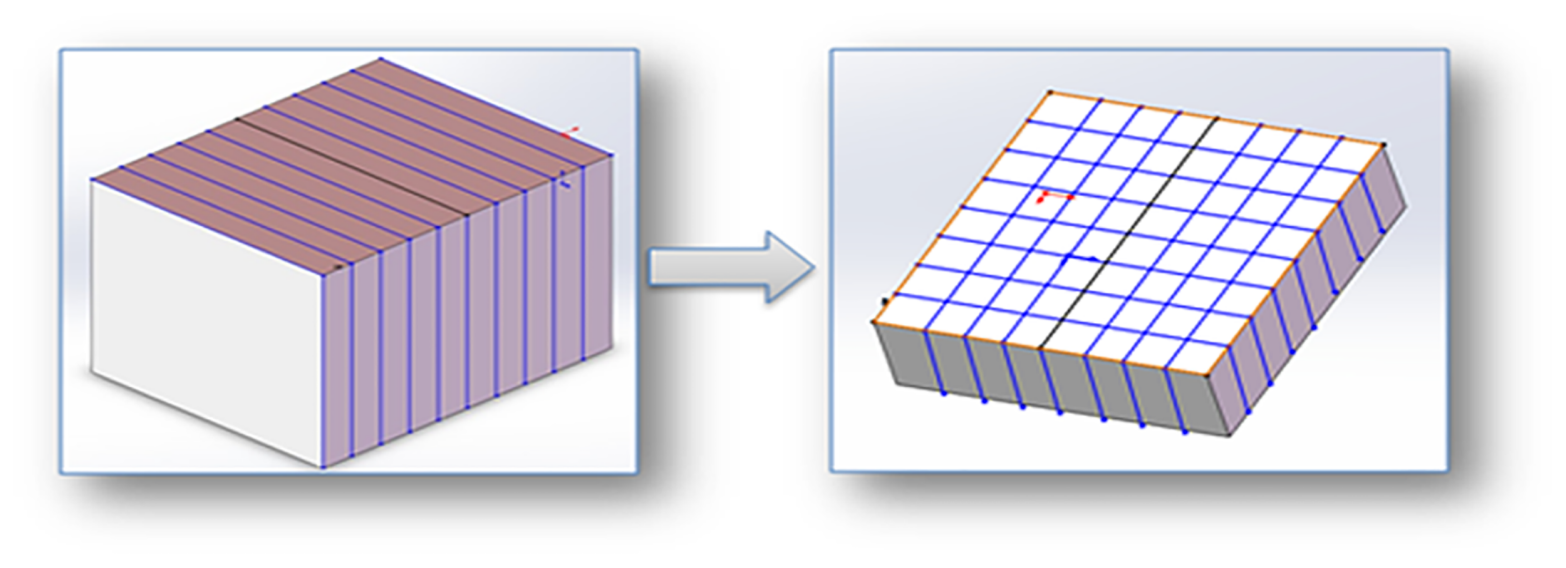
Data block partition.

**Fig 8:**
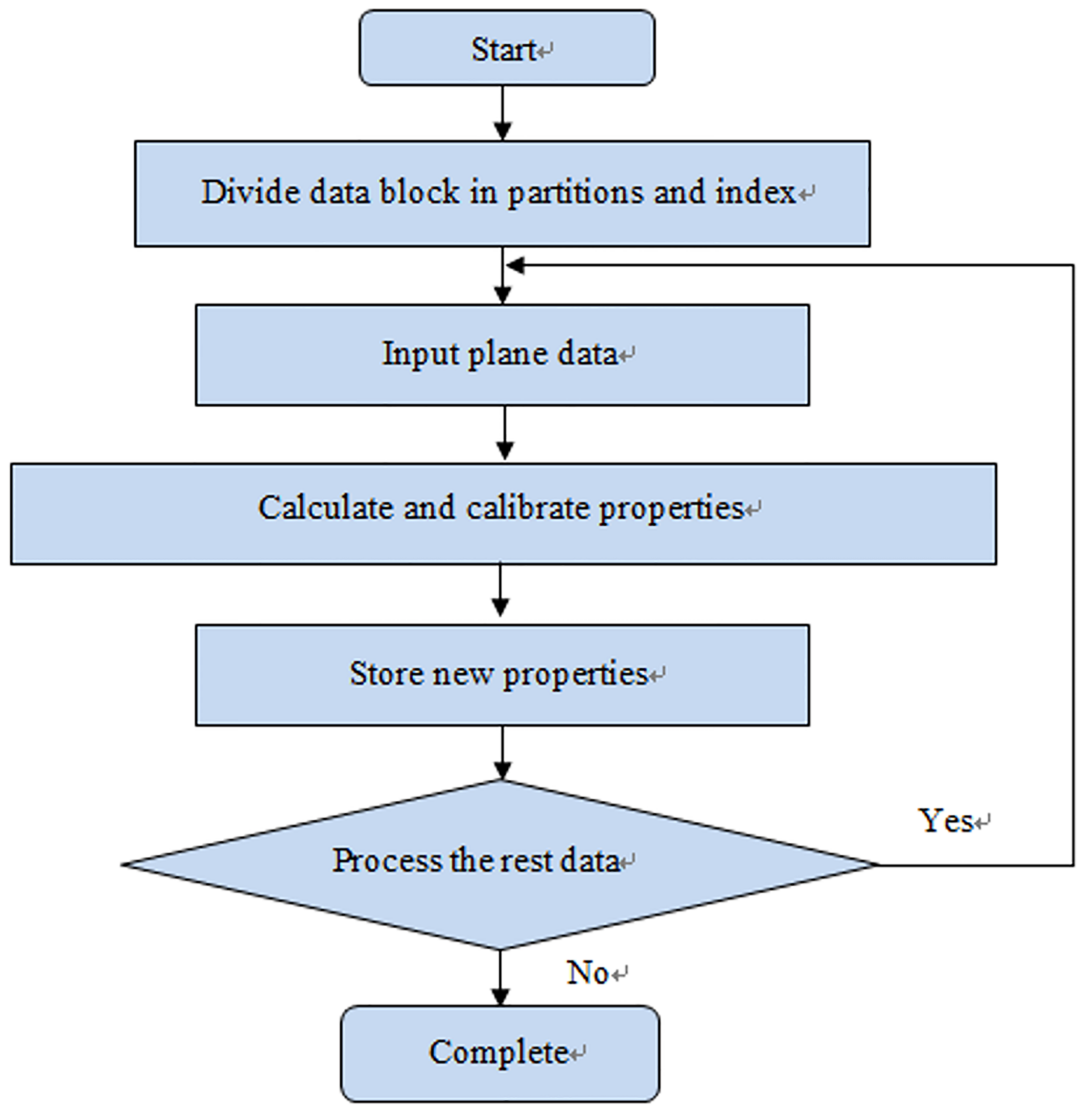
Workflow of data block partition.

One goal of model calibration is to convert the model and properties into a format that can be read by computers. When there are multiple geological mesh models, properties cannot be read at once. Therefore, the model was calibrated block by block. As shown in Fig 7, the whole model was first divided into slices, and then each slice was divided into columns to improve the efficiency of data reading. Fig 8 demonstrates the procedure of the dividing models.

After the initial rock mechanical model has been calibrated, it is necessary to save a new model and export property data. In this study, the mesh coordinates and rock mechanics properties were saved in text format, and the Young’s modulus and Poisson’s ratio were saved at different physical locations.

### Analysis

Based on the frequencies *t*_*1*_ and *t*_*2*_, the corresponding *p*_*1*_ and *p*_*2*_ can be calculated, which are the probability of the wellbore Poisson’s ratio and initial Poisson’s ratio at the same Poisson’s ratio value. Therefore, under each Poisson’s ratio, there will be two sets of probability. Part of the values are shown in Table 1.

**Table 1:** Probability of selected Poisson’s ratio.

Poisson’s ratio	Probability of Poisson’s ratio at the wellbore	Probability of Poisson’s ratio in the initial model	Error
0.246	0.001572	0.000422	-0.001150
0.248	0.003510	0.000759	-0.002750
0.250	0.004105	0.004470	0.000365
0.252	0.005707	0.006241	0.000534
0.254	0.010834	0.008686	-0.002150
0.256	0.014374	0.012565	-0.001810
0.258	0.021027	0.023022	0.001995
0.260	0.033417	0.032889	-0.000530
0.262	0.061036	0.068899	0.007863
0.264	0.097047	0.123630	0.026582
0.266	0.127001	0.166976	0.039975
0.268	0.133669	0.153061	0.019392

**Fig 9:**
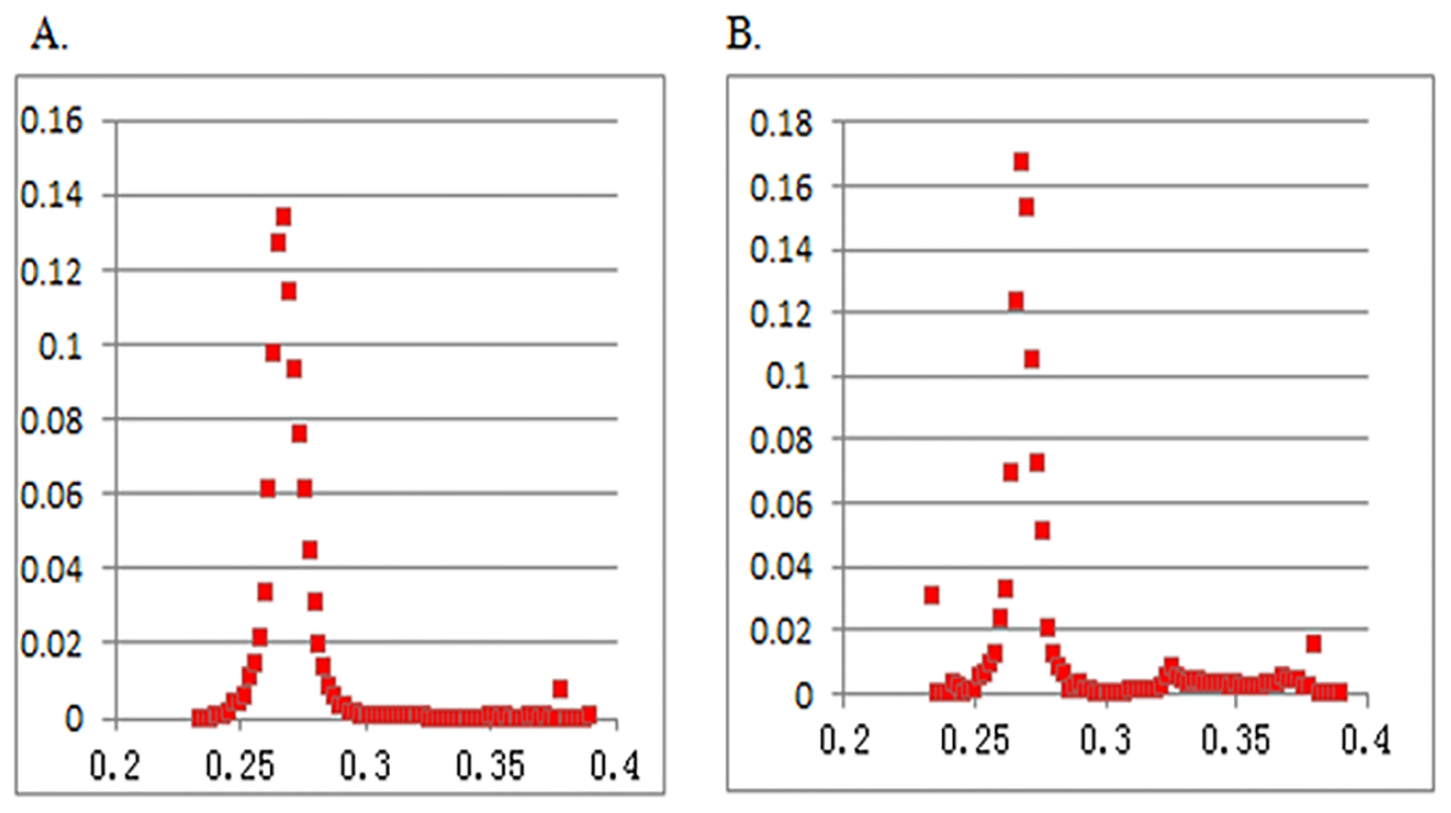
Probability of Possion’s ratio. (A) Probability of Possion’s ratio at wells. (B) Probability of Possion’s ratio in initial model.

Two sets of probability are plotted in Fig 9. The left picture is the probability distribution of the wellbore Poisson’s ratio, and the right picture is the probability distribution of the initial Poisson’s ratio. The vertical coordinates represent the probability, whereas the horizontal coordinates represent the Poisson’s ratio value.

**Fig 10:**
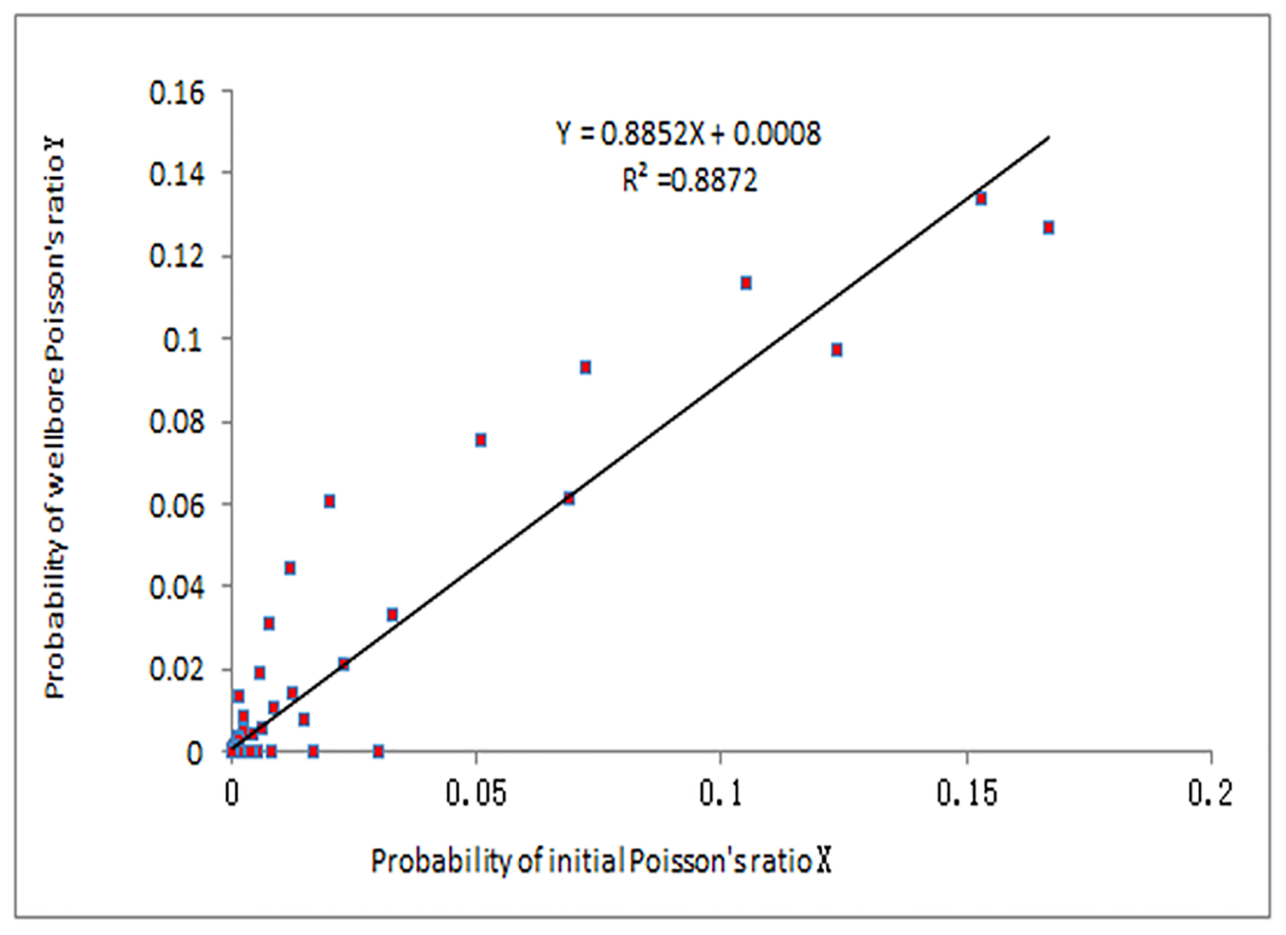
Regression analysis of Poisson’s ratio.

The linear relationship between the two sets of probabilities was established with a regression. As shown in Fig 10, the correlation coefficient is 0.8872. Assuming the slope in the linear regression equation is *K*, *k*_*1*_ can be solved by $k_{1}=K/\sqrt{\Delta \sigma }$, and then, k_2_ can be solved by $m_{1}=k_{1}*\sqrt{\Delta \sigma }*m_{2}+k_{2}*\Delta m$.

With *k*_*1*_ and *k*_*2*_ having been solved (*k*_*1*_ = 0.8763, *k*_*2*_ = -2.33*10^3^), the constraint model of rock mechanics parameters was established as follows:

$$Pois_{1}\left(x\right)=0.8763*\sqrt{\Delta \sigma }*Pois_{2}\left(x\right)-2.33*10^{3}*\Delta m$$ (8)

The accuracy of the initial Poisson’s ratio model largely increases after the calibration, and the initial Young’s modulus model can also be calibrated in the same way. The workflow of the model calibration can be summarized as follows:

1. Collect all the data related to rock mechanics properties from the wellbore and the initial model.
2. Find the maximum (*max*_*1*_, *max*_*2*_), minimum (*min*_*1*_, *min*_*2*_), average (*m*_*1*_, *m*_*2*_), variance (*σ*_1_, *σ*_2_) and frequency (*t*_*1*_, *t*_*2*_) of Young’s modulus and Poisson’s ratio.
3. If the differences (*m*_*1*_-*m*_*2*_) and (*σ*_1_-*σ*_2_) are within the acceptable error, stop the workflow, otherwise, continue. The error set in this study is 10%.
4. Apply correlation analysis between the probability of the wellbore Poisson’s ratio and the initial Poisson’s ratio given the same Poisson’s ratio value
5. Solve coefficients k_1_ and k_2_ using the regression method, and then, build a constraint model of rock mechanics parameters.

**Fig 11:**
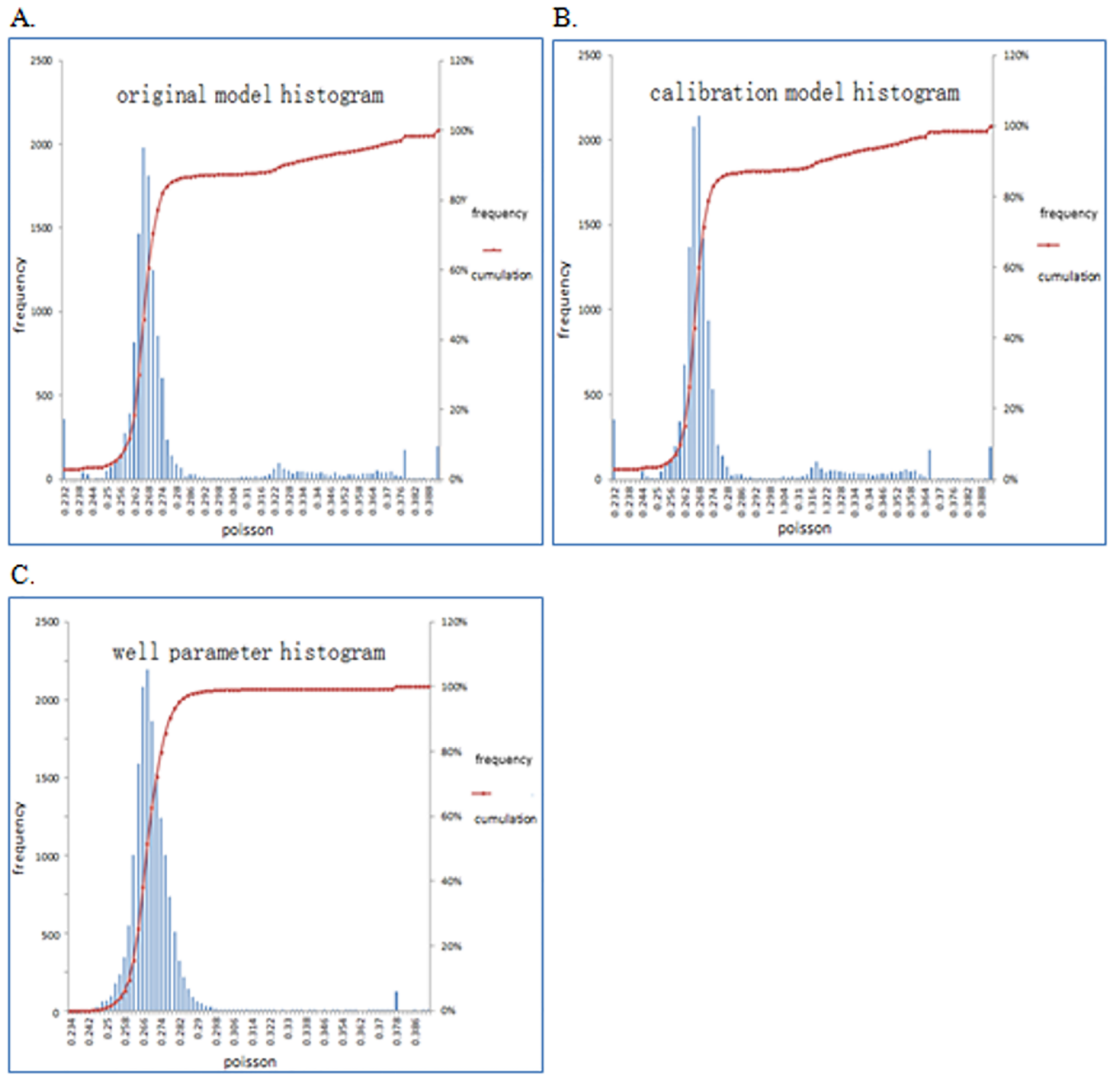
Histogram and cumulative probability distribution of Poisson’s ratio. (A) Probability distribution of initial model. (B) Probability distribution after calibration. (C) Probability distribution at wellbore.

Fig 11(b) shows the histogram of the Poisson’s ratio probability distributions after calibration. The probability distribution after calibration moves to the right to a certain extent compared with the probability distribution of the initial model in Fig 11(a). The Poisson’s ratio corresponding to the maximum probability in Fig 11(b) is 0.288, which coincides with the Poisson’s ratio in Fig 11(c), which shows the histogram of the Poisson’s ratio probability distribution at the wellbore. In addition, the distributions of Poisson’s ratios on the left side of the maximum probability in Fig 11(b) and 11(c) seem to be similar. In total, the Poisson’s ratio probability distribution after calibration matches better with the distribution of the wellbore parameters than the initial model.

**Fig 12:**
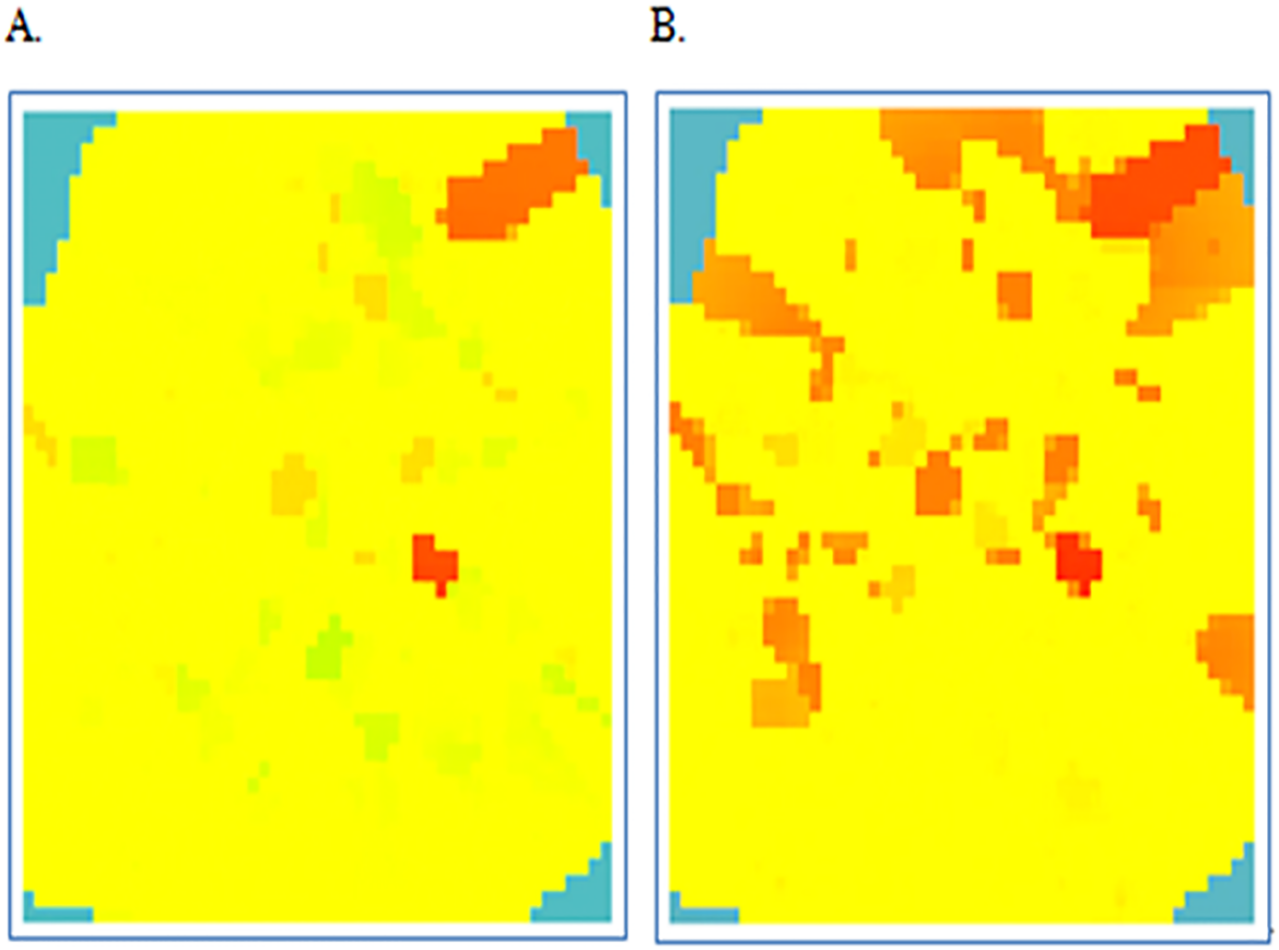
Poisson’s ratio distribution. (A) Poisson’s ratio distribution before calibration. (B) Poisson’s ratio distribution after calibration.

The Poisson’s ratio of the wellbore, the initial and calibrated models are summarized in Table 2. The maximum value, average, and variance at maximum probability of Poisson’s ratio after calibration are closer to those of the wellbore model. Therefore, the proposed method can calibrate the initial Poisson’s ratio effectively and improves the model accuracy. Fig 12 shows the models before and after the calibration, demonstrating the uneven distribution of the Poisson’s ratio obviously in this area, because more accurate geological information can be captured after calibration.

**Table 2:** Comparison of Poisson’s ratio in different models.

Poisson’s ratio of different situations	Poisson’s ratio at maximum probability	Minimum value	Maximum value	Average	Variance
Poisson’s ratio of initial model	0.2660	0.239627	0.458875	0.277743	0.033034
Poisson’s ratio after calibration	0.2680	0.242953	0.437032	0.276694	0.029242
Poisson’s ratio at wellbore	0.2680	0.239720	0.437426	0.269392	0.013350

**Fig 13:**
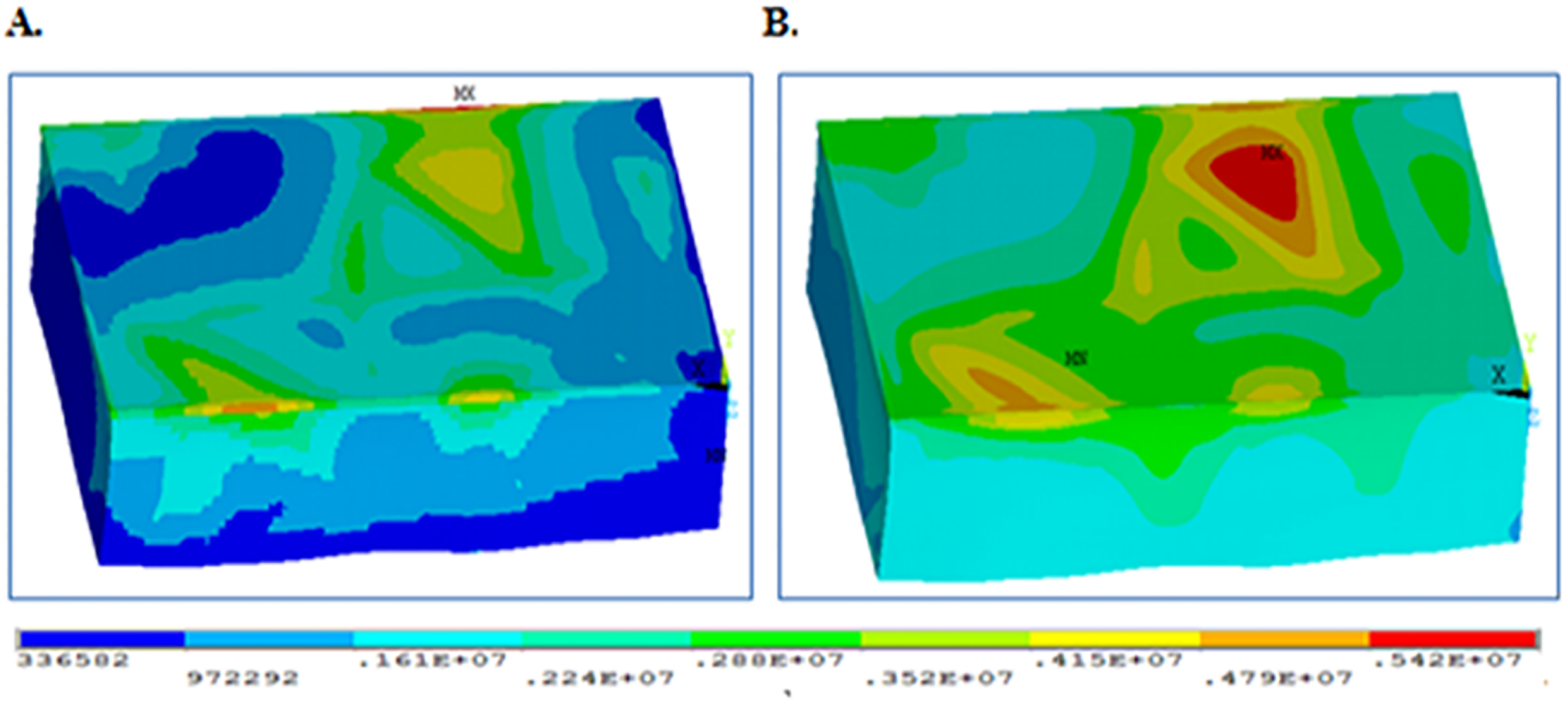
Three-dimensional geostress field distribution. (A) Minimum geostress distribution. (B) Maximum geostress distribution.

A data interface was developed to import the calibrated parameters and geological model to finite element software ANSYS, and the geostress was calculated successfully on a 3D finite model. Fig 13 shows the minimum and maximum geostress distribution.

## Conclusions

In this study, using conventional logging data and X-MAC logging data, rock mechanics parameters were calculated using a regression, a geological mesh model was built, and the initial three-dimensional rock mechanics model was constructed using Kriging. Based on this work, a new approach for calibrating three-dimensional models of rock mechanics parameters was proposed. Through analysis of the rock mechanics parameters of the wellbore and initial model, the optimal coefficients were calculated, which were important in building the constraint model. Then, the rock mechanics parameters could be calibrated using the constraints model; in this way, the accuracy of the initial mechanics model using the Kriging interpolation method was significantly improved.

## Supporting information

S1 File Partial data structure. Partial data structure used for developing the calibration model, including the interface of the calibration model (Fig 6).
(PDF)

S2 File EDITORIAL CERTIFICATE. The file certifies that the manuscript was edited for proper English language, grammar, punctuation, spelling, and overall style by one or more of the highly qualified native English speaking editors at American Journal Experts.
(PDF)

S3 File PAID ON 01/15/2017 9:25 PM. The file certifies that the revised manuscript has been paid to American Journal Experts.
(PDF)

S1 Table Test result analysis. Test result analysis proving the feasibility of the calibration system with different numbers of grids and giving the time spent during the test.
(DOC)

S2 Table Data for supporting results. Data is used for supporting the new calibration method in manuscript.
(XLS)
