# Supplementary material for: Research on calibrating rock mechanical parameters with a statistical method
Source: PLoS One. 2017 May 18;12(5):e0176215. doi: 10.1371/journal.pone.0176215 (PMC5436635; doi:10.1371/journal.pone.0176215)
Supplement: S1 File — Partial data structure used for developing the calibration model, including the interface of the calibration model (Fig 6). (PDF) [file pone.0176215.s001.pdf]

Partial data structure

```
class CDlgInput;
class CDlg_PropertyCalculator;
class Dlg_PropertyCalculator{
private:
        CString         m_Input;
        float           m_PropertyValue;
        long            m_namecount;
        CDlgInput       *m_pDlgInput;
        CDlgOutput      *m_pDlgOut;
        CDlg_PropertyCalculator    *m_pCDlgPropertyCalculator;
        virtual void    OnInputDlgChanged(CDlgInput *pInput);
public:
         bool   OnInit ();
        void    BracketsCalculator( float *p, CString str ,long iblock);
    }
```
