# Supplementary material for: Research on calibrating rock mechanical parameters with a statistical method
Source: PLoS One. 2017 May 18;12(5):e0176215. doi: 10.1371/journal.pone.0176215 (PMC5436635; doi:10.1371/journal.pone.0176215)
Supplement: S3 File — The file certifies that the revised manuscript has been paid to American Journal Experts. (PDF) [file pone.0176215.s003.pdf]

PAID ON 01/15/2017 9:25 PM

American Journal Experts

Send to:

American Journal Experts
601 West Main Street, Suite 102
Durham, NC 27701, United States
Phone: 1-919-704-4253
Fax: 1-919-287-2439
http://www.aje.com
Email: support@aje.com
Tax ID: 412141424

Invoice

Receipt code: **RYPI-4A7-0115211326**
Authors: Zhen Liu, Ye Guo, Shuheng Du, Gengyu Wu, Mao Pan
Title: The Research of Calibrating Rock Mechanical Parameters with Statistics Method
Submission date: January 15 2017, 09:25 pm

Invoice date	Description	Length	Time	Area of study	Price
January 15, 2017	Standard Editing	Standard (3501 - 6000 words)	7 days	Geophysics	$268.00
				Referral group #15PLOS discount	- $40.20
				Remaining balance	$0.00

PAYMENT METHOD(S): UnionPay
TERMS: Net 30 days. Online order
NOTES:
This invoice has already been paid, and is for your internal records only. Thank you for choosing American Journal Experts.
