# Supplementary material for: Research on calibrating rock mechanical parameters with a statistical method
Source: PLoS One. 2017 May 18;12(5):e0176215. doi: 10.1371/journal.pone.0176215 (PMC5436635; doi:10.1371/journal.pone.0176215)
Supplement: S1 Table — Test result analysis proving the feasibility of the calibration system with different numbers of grids and giving the time spent during the test. (DOC) [file pone.0176215.s004.doc]

**Table 1.** Test result analysis

| **Sequence** | **Grid numbers(million)** | **Time(second)** |
| --- | --- | --- |
| 1 | 1 | 6.898 |
| 2 | 10 | 64.045 |
| 3 | 1000 | 5980.260 |
